# Types and Mechanisms of Efflux Pump Systems and the Potential of Efflux Pump Inhibitors in the Restoration of Antimicrobial Susceptibility, with a Special Reference to *Acinetobacter baumannii*

**DOI:** 10.3390/pathogens13030197

**Published:** 2024-02-23

**Authors:** Kira M. Zack, Trent Sorenson, Suresh G. Joshi

**Affiliations:** 1 Center for Surgical Infections and Biofilms, Department of Surgery, College of Medicine, Drexel University, Philadelphia, PA 19104, USA; kmz58@drexel.edu; 2 Center for Surgical Infections and Biofilms, Drexel School of Biomedical Engineering, Science & Health Systems, Drexel University, Philadelphia, PA 19104, USA; trs336@drexel.edu

**Keywords:** *Acinetobacter baumannii*, antibiotics, carbapenem resistance, CRAB, efflux pump, efflux pump inhibitors, EPI, multidrug resistance, MDR, nosocomial infection, transferable antimicrobial resistance

## Abstract

Bacteria express a plethora of efflux pumps that can transport structurally varied molecules, including antimicrobial agents and antibiotics, out of cells. Thus, efflux pump systems participate in lowering intracellular concentrations of antibiotics, which allows phenotypic multidrug-resistant (MDR) bacteria to survive effectively amid higher concentrations of antibiotics. *Acinetobacter baumannii* is one of the classic examples of pathogens that can carry multiple efflux pump systems, which allows these bacteria to be MDR-to-pan-drug resistant and is now considered a public health threat. Therefore, efflux pumps in *A. baumannii* have gained major attention worldwide, and there has been increased interest in studying their mechanism of action, substrates, and potential efflux pump inhibitors (EPIs). Efflux pump inhibitors are molecules that can inhibit efflux pumps, rendering pathogens susceptible to antimicrobial agents, and are thus considered potential therapeutic agents for use in conjunction with antibiotics. This review focuses on the types of various efflux pumps detected in *A. baumannii*, their molecular mechanisms of action, the substrates they transport, and the challenges in developing EPIs that can be clinically useful in reference to *A. baumannii*.

## 1. Introduction

*Acinetobacter baumannii* is a Gram-negative aerobic coccobacillus that is mainly associated with nosocomial infections [1]. It commonly causes bloodstream, skin, urinary, and other soft tissue infections [1,2]. *A. baumannii* is currently on the priority lists of the healthcare-associated organizations, viz., the Center for Disease Control and Prevention (CDC), the National Institute of Health (NIH), and the World Health Organization (WHO), as it is a pathogen with a high propensity of acquiring and/or donating resistance genes to neighboring bacteria/microbiota [2,3,4]. *Acinetobacter* spp. are naturally competent, making it easier for them to acquire new plasmids and foreign DNA [5]. This ability allows *A. baumannii* to have a large associate genome, which helps it survive in a variety of different environments, including clinical settings [6,7]. In addition, *A. baumannii* naturally encodes efflux pumps, providing it with intrinsic resistance to antibiotics. *A. baumannii* constitutively expresses efflux pumps and has low membrane permeability, both of which allow it to survive a variety of antibiotics used [8]. In addition to overexpression of efflux pumps, other mechanisms of resistance are the production of carbapenemases, changes to penicillin-binding proteins, and the loss of outer membrane proteins. The genes for carbapenemases are usually carried on plasmids that are easily transmitted from cell to cell [5]. 

Studying *A. baumannii* has become increasingly important because of the rise in carbapenem-resistant *Acinetobacter baumannii* (CRAB) strains globally [1,9]. In different countries, the rate of resistance to carbapenem in *A. baumannii* ranges from 15% to 90% and is increasingly reported in nearly all countries [5,10,11]. Carbapenem is an important antibiotic in the treatment of nosocomial infections because of its potency, wide spectrum of activity, including both Gram-negative and Gram-positive bacteria, and lower levels of toxicity in comparison to other last-resort antibiotics [5]. Clinically, imipenem and meropenems are widely used broad-spectrum antibiotics in treating nosocomial infections [12,13]. A variety of different efflux pumps found in *A. baumannii* can use these antibiotics as substrates and export them out of the cell [5,8]. Since carbapenem is usually used as a last-resort antibiotic to treat critically infected patients, understanding and studying the control strategies of CRAB isolates are crucial to saving the lives of these patients.

## 2. Efflux Pumps

Efflux pumps work by transporting antibiotics out of the bacterial cell so that there is a low intracellular level of antibiotics, meaning that the antibiotics cannot reach their intended target [8,14]. Efflux pumps predate the use of antibiotics and have been shown to play vital roles in the physiology, pathogenicity, and metabolism of bacteria, which suggests that their primary role is not extruding antibiotics. Some of these activities include regulating nutrient and heavy metal levels, relieving cellular stress, and extruding toxins [15,16]. Also, efflux pumps may naturally export toxic substances, like bile, to help bacteria survive, invade, and colonize its host. BmrA, NorA, and MexAB-OprM extrude a variety of structurally unrelated antibacterial compounds [14,17]. Although efflux pumps, such as TetA and CmlA, are specific to certain antibiotics and provide intrinsic resistance to bacteria through chromosomal encoding [14,17], some pumps like MefA and MefE are encoded in transposons, and others, such as *OqxAB*, *qax*, *qepA*, and *tet*, can be carried on plasmids or integrons [14]. When exposed to antibiotics, bacteria often overexpress these efflux pumps, or the pumps may accumulate mutations, especially mutations in the regulatory genes, which allow them to efflux the antibiotics more efficiently. When efflux pumps are expressed constitutively, they often work together with other resistance mechanisms, like β-lactamases, to increase their resistance to antibiotics [18,19].

**Figure 1:**
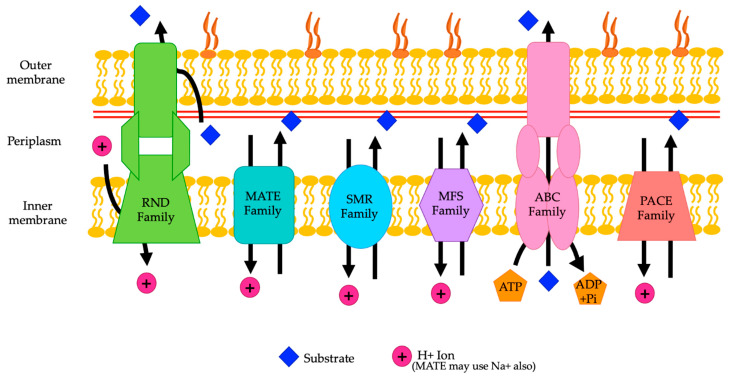
A representation of the various families of efflux pumps (RND, MATE, SMR, MFS, ABC, and PACE) and their locations in the membrane of *A. baumannii*. The direction of movement of the substrates is shown by the arrows (adapted from Verma et al. 2021 and Kornelsen and Kumar 2021).

*A. baumannii* can carry a variety of different genes for efflux pumps, which contributes to resistance to numerous antibiotics. *A. baumannii* can carry efflux pump genes from the resistance nodulation division (RND) family, ATP binding cassette (ABC) transporter family, multidrug and toxin extrusion (MATE) family, major facilitator superfamily (MFS), small multidrug resistance (SMR) family, and proteobacterial antimicrobial compound efflux (PACE) family [8,14] (Figure 1) (Table 1).

### 2.1. RND Family Efflux Pumps

RND efflux pumps are found in the domains of Eubacteria, Archaea, and Eukarya [8]. Clinically, they are the most important in Gram-negative bacteria [20]. The general structure of RND transporters comprises a tripartite complex, which includes an inner RND membrane protein, an outer membrane protein (OMP), and a membrane fusion protein (MFP) (also known as a periplasmic adapter protein; PAP) [8,21]. This tripartite pump extends across both the inner and outer membranes of bacteria, which is different than those of other families of efflux pumps since they generally only transport substrates across a single membrane. The MFP connects the RND protein and OMP [8]. There are 12 predicted transmembrane segments (TMSs) in the RND protein. Between TMS1 and TMS2 and TMS7 and TMS8, there are two long loops [22]. The OMP is a trimer that creates a continuous channel that crosses the periplasmic space and the outer membrane, which allows solvents to pass through it [23]. RND transporters are trimers that contain interdependent protomers. These protomers cycle through loose, tight, and open conformations and have proximal and distal binding pockets that have a variety of substrates they can bind [24]. In the RND efflux pump family, the proximal binding pocket (PBP) is conserved, except for residues 660–688 (using the numbering from AdeJ in *A. baumannii*). The region that is not conserved forms the bottom of the PBP and covers the flexible loop (F-loop) [24]. The MFP plays a role in the stabilization of the OMP by bringing the inner and outer membranes closer together [23]. 

A proton motive force is used by RND family efflux pumps to extrude substrates. RND family efflux pumps are proton antiporters and exchange one hydrogen ion for one molecule of a substrate. An increasing number of studies have shown that some pumps can directly efflux substrates from the cytoplasm [8]. Generally, the RND family of efflux pumps transports a variety of substrates from the periplasm. Mutations in the regulators of RND family efflux pumps allow for the overexpression of these efflux pumps. These mutations are widely found in clinical isolates due to selective pressure caused by using antibiotics. In addition, like many other types of efflux pumps, it is believed that RND efflux pumps play a role in normal physiological functions in many genera of bacteria, and that may be why they are such prevalent RND transporters and have a wide range of substrates.

Multidrug-resistant strains of *A. baumannii* commonly carry RND transporters, which are chromosomally encoded. Their exact physiological role in *A. baumannii* is unknown, and further studies are needed. It is believed that RND efflux pumps may naturally play a role in virulence and oxidative and nitrosative stress relief. In *A. baumannii*, the role of RND efflux pumps in multidrug resistance is being established. On average, there are an estimated 14 operons that code for RND efflux pumps in the *A. baumannii* genome, but these may vary from strain to strain.

**Table 1:** A representative table showing the efflux pumps and their regulators and substrates.

Family	Efflux Pump Name	Regulator(s)	Encoded	Substrates
RND	AdeABC	AdeRS and BaeSR [25,26]	Chromosomally [17,27]	Aminoglycosides, fluoroquinolones, β-lactams, chloramphenicol, trimethoprim, erythromycin, tetracyclines, netilmicin, gentamicin, macrolides/lincosamides, benzalkonium chloride, deoxycholate, nalidixic acid, methyl viologen, SDS, EtBr, and tigecycline * [21,23,25,28,29,30,31]
	AdeDE	Unknown [22]	Chromosomally [29]	Meropenem, erythromycin, chloramphenicol, ceftazidime, tetracycline, amikacin, ciprofloxacin, EtBr, and rifampin [22]
	AdeFGH	AdeL, ddrR, and abaI [32,33,34,35]	Chromosomally [27]	Trimethoprim, chloramphenicol, clindamycin, tetracycline-tigecycline, sulfonamides, fluoroquinolones, EtBr, SDS, safranin O, and acridine orange [32]
	AdeIJK	AdeN and BaeSR [8,36,37]	Chromosomally [27]	β-lactams (meropenem and imipenem), tetracyclines, cephalosporins, fluoroquinolones, chloramphenicol, trimethoprim, rifampin, fusidic acid, erythromycin, lincosamides, novobiocin, acridine, pyonine, safranin, antifolates, minocycline, SDS, gentamicin, amikacin, ceftazidime, ciprofloxacin, ceftriaxone, trimethoprim-sulfamethoxazole, minocycline, and tigecycline [28,38]
	AdeXYZ	Unknown [8]	Chromosomally [27]	β-lactams, ciprofloxacin, tetracycline, rifampin, and chloramphenicol [29]
	AbeD	SoxR [39]	Chromosomally [27]	Benzalkonium chloride, ceftriaxone, tobramycin, rifampin, and gentamicin [39]
	AprAB	ArpR [40]	Chromosomally [27]	Aminoglycosides (amikacin and tobramycin) [40]
	AcrAB	AcrR and AnoR [41,42]	Chromosomally [27]	Acriflavine, tobramycin, and colistin [42]
	CzcABCD	CopRS/CuxRS [16]	Chromosomally [27]	Heavy metals (copper) [16]
MATE	AbeM	ppGpp [43]	Unknown	Fluoroquinolones, aminoglycosides, chloramphenicol, erythromycin, doxorubicin, daunorubicin, EtBr, rhodamine 6G, Hoechst 33342, acriflavine, DAPI, tetracycline, gentamicin, triclosan, acriflavine, EtBr, kanamycin, erythromycin, TPPCl, and trimethoprim [44]
	A1S_3371 [15]	Unknown [15]	Unknown [15]	Unknown [15]
SMR	AbeS	Unknown	Chromosomally [45]	Acridine orange, acriflavine, benzalkonium chloride, β-lactams, chloramphenicol, ciprofloxacin, deoxycholate, EtBr, tetraphenylphosphonium, erythromycin, novobiocin, and SDS [27,45]
	QacE	Unknown	Integron [27,46]	Quaternary ammonium compounds, cetrimide, chlorhexidine, and benzalkonium chloride [47,48]
	A1S_0710 [15]	Unknown [15]	Unknown [15]	Deoxycholate and SDS [29]
MFS	TetA	Unknown	Tn1721-like transposon [21]	Tetracycline and tigecyclines [49]
	TetB	Unknown	Plasmids [50]	Tetracycline and minocycline [44]
	CraA	Unknown	Chromosomally [21]	Chloramphenicol [51]
	CmlA	Unknown	AbaR1 resistance island [52]	Chloramphenicol [52]
	FlorR	Unknown	AbaR1 resistance island [52]	Chloramphenicol and florfenicol [27,52]
	AmvA	TetR-type regulator [53]	Chromosomally [21]	Erythromycin, various dyes, and various disinfectants [54]
	AbaF	Unknown	Chromosomally	Fosfomycin [55]
	AbaQ	Unknown	Unknown	Quinolones [56]
	EmrAB	EmrR [57]	Unknown	Colistin and polymyxins [58,59]
ABC	MacAB-TolC	BaeSR [60]	Unknown	Erythromycin and gramicidin; tigecycline [21,61]
	A1S_0536 [15]	Unknown	Unknown	Erythromycin [15]
	A1S_1535 [15]	Unknown	Unknown	Chloramphenicol and gentamicin [15]
PACE	AceI	AceR [62]	Unknown	Chlorhexidine and short-chain diamines [63,64]
	A1S_1503 [65]	Unknown	Unknown	Acriflavine [65]

* (needs further studies to confirm). SDS, sodium dodecyl sulphate; EtBr, ethidium bromide; TPPCl, tetraphenylphosphonium chloride; and DAPI, 4′,6-diamidino-2-phenylindole, a DNA-binding stain (adapted from Verma et al. 2021 and Kornelsen and Kumar 2021).

Overexpression of genes for different efflux pumps generally comes at some biological cost to the bacterial cell. This cost may be due to the extrusion of molecules that are necessary for the cell, excessive energy use by the increased number of efflux pumps, or the regulatory genes also regulating other genes that could affect the fitness of the bacteria [66]. Currently, the AdeABC, AdeDE, AdeFGH, AdeIJK, AdeXYZ, AbeD, AprAB, AcrAB, and CzcABCD efflux pumps have been characterized in *Acinetobacter* spp.

#### 2.1.1. AdeABC Efflux Pumps

The AdeABC efflux pump was the first efflux pump characterized in *A. baumannii* [23]. Clinically, AdeABC is the most important RND family efflux pump. Studies have shown that it plays an important role in multidrug resistance in *A. baumannii*. Compared to other efflux pumps, it is overexpressed in a large number of *A. baumannii* isolates, with approximately 80% of isolates expressing the *adeABC* operon [27,67,68]. The *adeABC* operon is chromosomally encoded. In this operon, the MFP is *adeA*, the RND protein is *adeB*, and the OMP is *adeC*. Sometimes, *adeC* is not detected in *A. baumannii* strains, but *adeAB* is still present and upregulated, which suggests that AdeAB can use a different OMP. This OMP is most likely AdeK, from the AdeIJK efflux pump, which is constitutively expressed [69,70]. A similar system is seen in other RND family efflux pumps. For example, the MexAB and MexXY efflux pumps in *P. aeruginosa* both use OprM as their OMP [8].

The AdeABC efflux pump is positively regulated by the AdeRS two-component system (TCS) and has a response regulator (RR) and a histidine kinase (HK). The HK in the AdeRS TCS is AdeS, and the RR is AdeR. AdeS is part of the membrane and detects signals from the environment. When AdeS detects a signal, it auto-phosphorylates and transfers the phosphoryl group to AdeR to continue the signal cascade and activate the transcription of *adeABC*. AdeS senses saline stress, pentamidine, and other environmental stressors, causing the upregulation of genes in the *adeABC* operon [71]. The regulatory genes *adeR* and *adeS* lie just upstream of *adeA* and are transcribed in the opposite direction of the *adeABC* operon. Studies have shown that *adeS* is necessary for the expression of AdeABC [25]. AdeR is 228 amino acids long, and AdeS is shorter. The C-terminal of AdeS is highly conserved, with three D boxes, H, N, G1, F, and G2 boxes, and one K box of the regulator [72]. BaeSR is another TCS that regulates the expression of the AdeABC efflux pump, and it activates the *adeAB* operon, senses osmotic stress, and plays a role in tigecycline resistance [26]. It is not clear whether the AdeSR and BaeSR systems interact with each other. The *adeABC* operon may also be regulated by ppGpp [43]. In addition, it is reported that the expression of *adeABC* and *adeRS* increases when there is low iron in the environment and when human serum albumin is present [73,74]. In clinical isolates, there are often mutations in the AdeRS TCS that lead to the overexpression of the AdeABC efflux pumps [72]. Most commonly, mutations inactivate the phosphatase activity of AdeS. Specifically, this mutation is a G_103_D mutation in the histidine kinase, adenylyl cyclase, methyl-accepting protein, and phosphatase (HAMP) linker domain between the DHp domains and the sensor and a mutation in T_153_M in the H box. Because of this mutation, AdeS can no longer dephosphorylate AdeR, leading to continuous overexpression of the AdeABC efflux pump. AdeR can also have mutations, but these have a smaller effect and generally alter the stability of the protein. These mutations most commonly affect the effector binding pockets and phosphorylation sites. The most common mutations found include a D_20_N mutation at the phosphorylation site in one of the three D boxes, an A_91_V mutation in the signal receiver domain, and a P_116_L mutation at the first amino acid residue of the helixα5 that is required for the continuation of the phosphorylation-triggered signal [25,72]. Single point mutations in *adeS* (T_153_M) and *adeR* (P_116_L) can cause constitutive expression of the AdeABC efflux pump, resulting in spontaneous gentamicin resistance [25].

**Figure 2:**
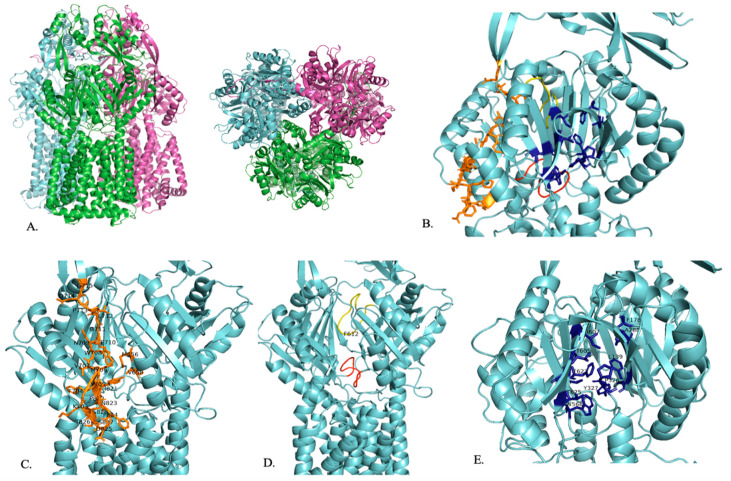
A representation of the structure of AdeB. (**A**) Trimeric structure of AdeB from two viewpoints. (**B**) The proximal binding site (orange), F-loop (red), G-loop (yellow), and distal binding site (dark blue) of AdeB are shown within one segment of the trimer. (**C**) The proximal binding site (orange) with residues that play a role in substrate entry and recognition is labeled. (**D**) F-loop (red) and G-loop (yellow) with their conserved F612 residue labeled. (**E**) Distal binding site (dark blue) with residues that play a role in substrate binding is labeled [75].

AdeB is a homotrimer, with each subunit consisting of 12 transmembrane helices and six periplasmic subdomains (PN1, PN2, PC1, PC2, DN, and DC) (Figure 2A) [75]. Subdomains PC1 and PC2 make up the periplasmic cleft. Each AdeB protomer contains an extrusion channel [76]. The transmembrane domains of AdeB can be in associated forms, where they are a trimer, or in dissociated forms, where they can be in dimer plus monomer or monomer plus monomer plus monomer configurations. AdeA is a hexameric channel that interacts with AdeB, which controls the opening and closing of AdeA through its rotations [76].

AdeB has a multidrug binding site that has three clefts: a periplasmic cleft, a proximal site, and a distal site (Figure 2B). Carbapenem enters the pump in its open form at the periplasmic site [77]. Like the other RND family efflux pumps, the substrate enters the periplasmic clefts, moves to the proximal binding site, travels by the gate loop (G-loop), moves to the distal binding site, and then moves to the hydrophobic area of the binding site to then be exported out of the cell (Figure 2B) [76].

In the open conformation, there are shifts in the K703–715 and I821–S828 residues that allow carbapenem to enter AdeB at the periplasmic site [77]. The entrance site also includes residues M656, V658, M706, W708, and I821, which play a role in substrate recognition [76]. Then, carbapenem travels to the proximal site, located in the inner part of AdeB, where it binds (Figure 2C) [77]. The proximal site consists of the F-loop (P661–S670) and the G-loop (G609–N618) (Figure 2D) [77]. The F-loop sits at the bottom part of the proximal site and connects the entrance site and proximal site of the periplasmic cleft (Figure 2B,D) [76,77]. Carbapenem then uses the G-loop to move to the distal site, where it will be exported out of the cell (Figure 2B,D). In the closed state, the G-loop is positioned so that there is more room for the substrate at the distal site [77]. The F-loop and G-loop of AdeB are noted to be very flexible and have distinctly different conformations depending on whether the periplasmic cleft is in the open or closed conformation [76,78]. When the cleft is in the closed state, the F-loop and the G-loop block the proximal binding site. When the cleft is in the open state, the F-loop and the G-loop are positioned so that the proximal binding site is open [76]. The G-loop includes a conserved F612 residue that plays a role in substrate binding at the distal binding site and substrate export (Figure 2D) [76,78]. F612 and W610 are highly flexible and may help to push the substrate out of the efflux pump [76]. The AdeB binding site includes conserved phenylalanine residues that are important in substrate binding [78]. The distal binding site includes F136, L139, F178, A288, P326, Y327, W568, M570, T605, I607, F623, and T625, which are important for substrate binding (Figure 2E). The hydrophobic area of the binding site is critical for substrate binding and includes F178, F277, I279, I607, and W610 (Figure 2E). Residues T668, F669, and F612 stabilize the extrusion state of AdeB through hydrophobic interactions. A second flexible loop (residues 131 to 139) is also found in AdeB. The exit site of AdeB is formed by Q125 and Y749 [76].

AdeB has a slightly different mechanism of action than other RND family efflux pumps since it is believed that each AdeB trimer can independently export substrates. Also, a single trimer of AdeB can hold three substrates at a time, one at the proximal binding site, one at the distal binding site, and a hydrophobic region within the binding site [76]. 

Some of the substrates of the AdeAB efflux pumps are pentamides, which are dicationic compounds [71]. The AdeABC efflux pumps provide resistance to aminoglycosides, fluoroquinolones, β-lactams, chloramphenicol, trimethoprim, erythromycin, tetracyclines, netilmicin, gentamicin, macrolides/lincosamides, benzalkonium chloride, deoxycholate, nalidixic acid, methyl viologen, sodium dodecyl sulfate (SDS), and ethidium bromide (EtBr) [21,23,25,28,29]. Tigecycline may also be a substrate for AdeABC efflux pumps, but further experiments are needed to confirm this [21,28,31]. The AdeABC efflux pump is strongly associated with carbapenem resistance as compared to other efflux pumps that *A. baumannii* carries [5]. When carbapenem-hydrolyzing oxacillinases (OXAs) are present and the AdeABC efflux pump is overexpressed, *A. baumannii* has a high level of resistance to carbapenem [67,68]. The overexpression of *adeABC* also plays a role in biofilm formation, natural transformation, plasmid transfer, competence, iron acquisition, motility, and virulence, which suggests that the AdeABC efflux pump may have a variety of different physiological functions [28,69,79,80]. 

#### 2.1.2. AdeDE Efflux Pumps

The AdeDE efflux pump system is not well characterized, and the mechanisms for how it regulates its expression are not clear. More research is needed to understand the mechanisms and movement of this efflux pump. It is found in *Acinetobacter pittii*/*A. baumannii* (GDG3), and the gene *adeE* has also been reported in *Acinetobacter lwoffii* [22]. AdeD is the MFP, and AdeE is the RND transporter protein. The AdeDE efflux pump does not have a gene that encodes the OMP in its operon [22]. It has been found to increase resistance to meropenem, erythromycin, chloramphenicol, ceftazidime, tetracycline, amikacin, ciprofloxacin, rifampin, and EtBr [22]. It is believed that adeDE uses a proton motive force to export its substrates and is chromosomally encoded [29].

#### 2.1.3. AdeFGH Efflux Pumps

The AdeFGH efflux pump is chromosomally encoded but not constitutively expressed. Therefore, it does not play a role in intrinsic resistance and must be overexpressed to cause resistance [27]. In a study by Coyne et al., the AdeFGH efflux pump was detected in 90% of *A. baumannii* strains examined [32]. The AdeFGH efflux pump is regulated by AdeL, a LysR-type transcriptional repressor (LTTR), that lies immediately upstream of the *adeFGH* operon. AdeL has a N-terminal DNA-binding domain with a helix-turn-helix motif between residues 11 and 32. The C terminus of AdeL has a co-factor binding domain. Based on the available intergenic sequencing results, the promoters of *adeL* and *adeFGH* may be overlapping since there is a typical LTTR box motif (TTA-N_7_-TAA) present, which is where DNA binding by LTTRs occurs.

In clinical isolates, mutations in AdeL usually cause multidrug resistance because these mutations lead to the overexpression of AdeFGH [72,81]. Mutations in AdeL are most often found in the C-terminal regulators, which leads to the constitutive expression of the *adeFGH* operon [28]. Some of these mutations in the C-terminal domain of AdeL include a T_319_L substitution and/or loss of the last 11 amino acid residues due to a deletion. Both mutations can create issues with RNA polymerase interacting with the regulator and oligomerization. A V_139_G substitution may cause AdeL to act even when there is no signal, because this region plays a role in signal recognition [27]. Human serum albumin in the environment also increases the overexpression of AdeFGH efflux pumps [73]. The AdeFGH efflux pump may also be overexpressed when DNA is damaged. This overexpression of AdeFGH may be caused by *ddrR,* a DNA-damage inducible gene that was first identified in *Acinetobacter baylyi* but has now been shown to be conserved in *Acinetobacter* spp. [33,34]. Canadian isolates of *A. baumannii* more commonly overexpress the AdeFGH efflux pump than the AdeABC and AdeIJK efflux pumps [82]. This is unusual, as overexpression of AdeFGH has the largest negative effects on bacterial fitness in comparison to overexpression of the other two RND efflux pumps [66]. Also, previous studies have found that there is a link between *abaI*, a quorum sensing gene, and *adeG*. When *abaI* and *adeG* are overexpressed, there is increased biofilm formation [35]. These studies also suggested that there is one directional cross regulation between *adeFGH* and *adeAB,* but the exact mechanism is unknown. Further research is required to understand the mechanisms and movement of this efflux pump. When this efflux pump is overexpressed, there is increased resistance to trimethoprim, chloramphenicol, clindamycin, tetracycline–tigecycline, sulfonamides, and fluoroquinolones, and other substrates of the AdeFGH efflux pump include EtBr, SDS, safranin O, and acridine orange [32].

#### 2.1.4. AdeIJK Efflux Pumps

Currently, the chromosomally encoded, constitutively expressed AdeIJK efflux pump has been found in all strains of *A. baumannii* and is also believed to play a major role in antibiotic resistance [27,28,38,67]. In addition, AdeIJK homologues can be found in many *Acinetobacter* spp., wherein there is >90% amino acid residue similarity in the *Acinetobacter calcoaceticus*/*Acinetobacter baumannii* complex, which includes *A. calcoaceticus*, *A. pitti*, *A. baumnannii*, and *A. noscomialis* [36]. The prevalence of the AdeIJK pump suggests that it plays an important physiological role in *A. baumannii* and the entire *Acinetobacter* spp., in addition to contributing to intrinsic multidrug resistance in this genus of bacteria [36,38]. Studies suggest that AdeIJK plays a role in the stability of the make-up of the membrane and the maintenance of lipid homeostasis, especially the export, biosynthesis, and turnover of lipids, and in motility [28,69,70,83]. In clinical isolates, overexpression of AdeIJK is uncommon, likely because overexpression of this pump is damaging to *A. baumannii* cells [28,36,38]. This level of expression is different than that of AdeABC and AdeFGH pumps, which can be greatly overexpressed (up to mid- to high double digits in lab strains). In lab strains of *A. baumannii* carrying AdeIJK, there can only be a 1-to-2-fold increase in expression with this pump [72].

The expression of the AdeIJK efflux pump is controlled by AdeN, a TetR-type transcriptional regulator that acts as a global regulator and controls the expression of virulence factors, biofilm formation, the response to environmental changes, and many other factors [8,36,37]. AdeN lies approximately 800 kb away from the *adeIJK* operon. This makes the AdeIJK system different than other RND efflux pumps, which usually have their regulators close to the genes they control the expression of. AdeN has a conserved DNA binding motif at its N terminus. Overexpression of the AdeIJK efflux pump is caused by mutations that lead to the truncation of the C terminus of AdeN [72]. The expression of AdeIJK is also controlled by BaeSr, a TCS [8,26,61]. BaeSR may contribute to crosstalk between AdeABC and AdeIJK since BaeSR plays a role in the expression of both efflux pumps [8]. In addition, ppGpp plays a role in the expression of AdeIJK, but the exact mechanism of action is unknown. Also, if human serum albumin is present in the environment, *adeIJK* and *adeN* are downregulated [73]. There may be other unknown mechanisms for controlling the expression of the AdeIJK efflux pump since it is rare to find high levels of overexpression of this pump [36].

**Figure 3:**
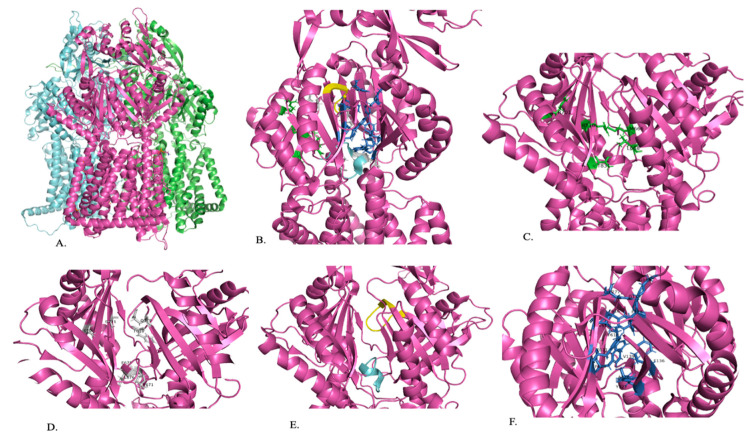
A representation of the structure of AdeJ. (**A**) Trimeric structure of AdeJ (**B**) The periplasmic cleft (green), proximal binding site (gray), F-loop (blue), G-loop (yellow), and distal binding site (dark blue) of AdeJ are shown within one segment of the trimer. (**C**) Periplasmic cleft (green) with residues playing a role in substrate specificity is labeled. (**D**) Proximal binding site (gray) with conserved residues is labeled. (**E**) F-loop (blue) and G-loop (yellow). (**F**) Distal binding site (dark blue) with residues that play a role in substrate binding is labeled [84].

AdeJ, AdeI, and AdeK are part of a tripartite system that works together to export antimicrobials. AdeJ is trimeric and is located in the inner membrane of the system [84]. It recognizes substrates and generates the proton motive force needed to export substrates. Residues D407, D408, K953, and T989 in the transmembrane domain of AdeJ play a role in proton relay and are conserved. AdeI is the periplasmic membrane fusion protein. AdeK is the outer membrane channel. AdeJ is a homotrimer, with each protomer having six subdomains in the periplasmic region (Figure 3A). Four of these subdomains (PN1, PN2, PC1, and PC2) make up the portal domain, and the other two (DN and DC) make up the docking domain. Subdomains PC1 and PC2 create a cleft where the substrate can enter and bind the efflux pump. This cleft is surrounded by M666, L668, R701, R718, and T831, which are important in substrate specificity (Figure 3C). This cleft can switch between one open and two closed (extrusion state and resting state) conformations. There is only one open periplasmic cleft at a time. In the open conformation, there is a channel leading from the open cleft to the periplasmic domain that allows substrate exposure to the binding site. In the extrusion site, the cleft is closed, and there is a channel running perpendicular to the membrane surface in the periplasmic domain. In the resting state, the cleft is closed and there is no channel [78].

Once the substrate has entered the cleft, it is guided by the F-loop to the proximal binding site (Figure 3B). The F-loop helps to create the bottom portion of the proximal binding site (Figure 3B,E). Residues 671 to 680 are conserved in the F-loop and connect the entry cleft to the proximal binding site (Figure 3E). The proximal binding site consists of at least 22 residues, with S79, Q579, F618, E675, L676, G721, and G21 being conserved in AdeJ, AdeB, and AcrB, and M575 and R718 being conserved in AdeJ and AcrB (Figure 3D). The substrate will then pass through the G-loop to the distal binding site, where it will be exported out of the cell (Figure 3B,E). The binding site includes amino acids F136, V139, F178, G179, G180, F277, A326, Y327, GF611, V613, F616, F618, and F629, which anchor the substrate. Residues 615 to 624 of the G-loop are conserved and separate the proximal and distal substrate binding sites (Figure 3B,E). The distal binding site includes six residues (AdeJ F136, F178, Y327, V618, and F629) that are conserved between AdeJ, AdeB, and AcrB. AdeJ F277, M57, and F616 play an important role in substrate recognition and are conserved in AdeB and AcrB, respectively (Figure 3F). A hydrophobic patch made of F178, F277, V613, and F616 is noted in the distal binding site of AdeJ. It is important for substrate binding and export. It is noted that the general mechanism of action for recognizing substrates is likely due to aromatic and hydrophobic interactions [78].

The AdeIJK pump provides resistance against amphiphilic compounds [38]. These include β-lactams, tetracyclines, cephalosporins, fluoroquinolones, chloramphenicol, trimethoprim, rifampin, fusidic acid, erythromycin, lincosamides, novobiocin, acridine, pyonine, safranin, antifolates, minocycline, and SDS [28,38]. Studies have also shown that the AdeIJK pump provides resistance to gentamicin, amikacin, the β-lactams meropenem and imipenem, ceftazidime, ciprofloxacin, ceftriaxone, and combination trimethoprim–sulfamethoxazole in some strains of *A. baumannii*. The AdeABC and AdeIJK efflux pumps work together in a more-than-additive manner to provide resistance to minocycline, tetracycline, and tigecycline [38].

#### 2.1.5. AdeXYZ

The AdeXYZ efflux pump is found in *A. baylyi* and *Acinetobacter* GDG3, and *adeY* has also been reported in *Acinetobacter lwoffii* [22]. This pump needs to be further characterized to understand its regulation [8]. AdeXYZ shares 93% nucleotide identity and 99% amino acid identity with AdeIJK, but they have been shown to be separate subfamilies of the RND efflux pumps [24,85]. The amino acid sequences of AdeJ and AdeY are identical, except for a single insertion after position 602 (position based off AdeJ found in *A. baumannii*) in AdeY. This amino acid is in a surface protein, so it does not affect substrate binding. There is a high conservation of the residues within the PBP of AdeJ and AdeY, including residues 660–688 (position based off AdeJ found in *A. baumannii*). This is a significant difference between these efflux pumps and AdeB and AcrB. Notably, both AdeJ and AdeY have V573, which is not present in the other RND family efflux pumps. It is also significant that R718 is conserved in AdeJ, AdeY, and AcrB but not AdeB. The distal binding pockets (DBPs) of AdeJ and AdeY have four variable residues. Three of these residues (A46, Q91, and T128) are located at the back of the DBP and are likely responsible for the differences in substrate binding. Residue AdeJ F611 plays a critical role in ligand binding in the DBP and is also conserved in AdeY and AcrB (F610) [24]. Across the DBPs of AdeJ, AdeY, adeb, and AcrB, F136, F178, Y327, V613, F618, and F629 (position based off AdeJ found in *A. baumannii)* are universally conserved. Residue Y327, which is located at the bottom of the DBP, in AdeJ and AdeY show variation. This residue has direct contact with M575 and T679 in the PBP, both of which are also variable residues. The DBP is separated from the PBP by a flexible G-loop (residues 613–624 in AdeJ in *A. baumannii*). The G-loop has a conserved F618 residue in AdeJ, AdeY, and AdeB that plays a role in substrate binding in the DBP. Its substrates are β-lactams, ciprofloxacin, tetracycline, rifampin, and chloramphenicol. It is believed that the AdeXYZ efflux pump uses a proton motive force to extrude its substrates [29].

#### 2.1.6. AbeD

AbeD is found *A. baumannii* AYE [39]. It was not found with the accompanying OMP or PAP genes, like the rest of the RND efflux pumps are. More research is required to further characterize this efflux pump, determine what transporter it works with, and understand the mechanisms and movement of this efflux pump. AdeD is directly regulated by SoxR, a part of the SoxSR TCS, which normally regulates oxidative stress and plays a role in the tolerance of oxidative stress. AbeD has been found to increase resistance to benzalkonium chloride, ceftriaxone, tobramycin, rifampin, and gentamicin.

#### 2.1.7. ArpAB Efflux Pumps

ArpAB has been detected in *A. baumannii* AB5075 [40]. ArpA is a PAP, and ArpB is a RND transporter. ArpAB expression is controlled by ArpR, a TetR-type regulator [40]. More research is needed to understand the mechanisms and movements of this efflux pump. ArpB may be an aminoglycoside pump, since it provides resistance to amikacin and tobramycin. It is not known whether ArpAB has any other substrates. Also, in *A. baumannii*, the ArpAB efflux pump appears to play a role in virulence since it was seen to affect opaque/translucent colony phase variation in a number of studies [8,40,86].

#### 2.1.8. AcrAB Efflux Pumps 

AcrAB, found in *Acinetobacter noscomialis*, is homologous to ArpAB, which has been detected in *A. baumannii*. AcrAB expression is governed by AcrR. It acts as a repressor and lies upstream of the *acrAB* operon [41]. A quorum sensing regulator, AnoR, seems to activate *acrAB’s* expression, and interestingly, the AnoIR quorum sensing system is repressed by AcrR. No interplay between the efflux pumps and quorum sensing systems has been observed in *Acinetobacter* spp., unlike in other bacteria, like *Pseudomonas* spp. [42]. The AcrAB efflux pump works with TolC to export substrates out of the cell. AcrA is a periplasmic linker protein. TolC is an outer membrane channel. AcrB is a homotrimer, with each of its subunits comprising 12 transmembrane helices and two periplasmic regions [87]. 

AcrB shares highly conserved regions with AdeB and is so structurally similar to AdeB that their binding pockets can be directly compared. Residue F610 in AcrB plays a critical role in ligand binding in the DBP and is also conserved in AdeY and AcrB (F611) [24]. R717 at the entrance of the periplasmic cleft plays a role in substrate specificity [78]. The proximal binding site of AcrB contains at least 22 residues, and the distal binding site includes at least 23 residues. The distal binding site includes a hydrophobic patch similar to the one in Ade that is important in substrate stabilization [78,88]. This hydrophobic region includes F178, I277, V612, and F615. Binding is also influenced by 15 nearby hydrophobic and 11 polar or charged residues [88]. The AcrB binding site includes conserved phenylalanine residues that are important for substrate binding [78]. AcrB has a similar mechanism of action to those of the other RND family efflux pumps. The substrate binds in the proximal binding pocket, moves to the DBP, and then detaches to be exported out of the cell [88]. The AcrAB efflux pump increases resistance to acriflavine, tobramycin, and colistin.

#### 2.1.9. CzcABCD Efflux Pumps

The CzcABCD efflux pump has yet to be characterized in detail to determine the mechanism of function and its substrates. The CzcABCD efflux pump is composed of an operon, which consists of five genes. It codes for a hypothetical protein, then czcB, czcA, and czcD, and codes for the PAP, RND, and OMP, respectively. CzcABCD allows for tolerance to heavy metals, especially copper. The expression is regulated by CopRS/CuxRS TCSs in response to copper in the environment [16].

### 2.2. MATE Family Efflux Pumps

MATE family efflux pumps have been found in the Archaea, Eukarya, and Eubacteria domains of life [8]. MATE efflux pumps function as antiporters. They pump out cations in exchange for hydrogen or sodium ions. These pumps contain 12 transmembrane helices [8]. MATE family efflux pumps move between open and closed conformations, where the substrate binding site is only exposed in the open conformation [89]. 

*A. baumannii* carries the AbeM efflux pump from the MATE family [44]. It is 447 amino acids long with multiform hydrophobic regions [29]. The most common substrates for the AbeM efflux pump are fluoroquinolones, aminoglycosides, chloramphenicol, erythromycin, doxorubicin, daunorubicin, EtBr, rhodamine 6G, Hoechst 33342, acriflavine, 4′,6-diamino-2-phenylindol (DAPI), tetracycline, gentamicin, triclosan, acriflavine, EtBr, kanamycin, erythromycin, tetraphenylphosphonium chloride (TPPCl), and trimethoprim [44]. Studies on *E. coli* have shown that the AbeM efflux pump provides increased resistance to norfloxacin, ciprofloxacin, and, to a lesser extent, ofloxacin [44]. In *E. coli*, the AbeM efflux pump provides a minor increase in resistance to triclosan, trimethoprim, chloramphenicol, and some aminoglycosides. The expression of the AbeM efflux pump may be controlled by levels of ppGpp, which also affects the expression of the AdeIJK and AdeABC efflux pumps, which belong to the RND family [43]. Another MATE family efflux pump found in *A. baumannii*, A1S_3371, plays a role in motility and virulence [15]. More research is required to determine their role in antimicrobial resistance, their mechanisms of action, and the details of their substrate binding sites. 

### 2.3. SMR Family Efflux Pumps

The efflux pumps in the SMR family are composed of small, integral inner membrane proteins [8]. These proteins have four transmembrane α-helix domains, and the proteins are likely to function as hetero- or homo-oligomers [8,29]. Efflux pumps within this family use a proton gradient to pump their substrates out of a cell. The substrates of SMR family efflux pumps are lipophilic, cationic compounds, including quaternary ammonium compounds [90]. Therefore, these pumps allow bacteria to be resistant to many detergents and antiseptics.

In the *A. baumannii* genome, there are an average of four encoded SMR family efflux pumps [8]. Within the SMR family, AbeS is the only characterized efflux pump in *A. baumannii*. AbeS consists of a single 109 amino acid inner membrane protein and is the smallest efflux pump found chromosomally encoded in *A. baumannii* [45,91]. AbeS has four transmembrane helices. Residues Y3, A16, and A42 play a role in substrate recognition and coupling of hydrogen ion antiporters of substrates. A16 plays a critical role in this function. Y3 is located on the periplasmic side of the first transmembrane helix and is important in gating/coupling. A9 and A16 are part of the substrate binding site and are located at the periplasmic side of the first transmembrane helix, one helical turn above or below E13. The residues may play a role in excluding substrates based on size through small changes in the size of the substrate binding site. It appears that the substrate-binding site prefers hydrophobic compounds. A central loop within AbeS plays an important role in substrate transport. When it is immobilized, AbeS is unable to transport any substrates [91]. Its substrates are acridine orange, acriflavine, benzalkonium chloride, β-lactams, chloramphenicol, ciprofloxacin, deoxycholate, EtBr, tetraphenylphosphonium, erythromycin, novobiocin, and SDS [27,45]. It has a small role in decreasing the susceptibility of *A. baumannii* to these substrates. In the SMR family, there is also the QacE efflux pump in *A. baumannii*, which is encoded in an integron. Studies have shown that it is present in approximately 40% of *A. baumannii* strains [27,46]. Its substrates are quaternary ammonium compounds. In *A. baumannii,* the QacE efflux pumps reduce susceptibility to cetrimide, chlorhexidine, and benzalkonium chloride [47,48]. The QacE efflux pump also reduces the susceptibility of *Klebsiella pneumonia, Klebsiella aerogenes*, and *Proteus mirabilis* to quaternary ammonium compounds [8,92,93]. Another SMR family efflux pump, A1S_0710 in *A. baumannii,* has been shown to play a role in motility and virulence [15]. Its substrates are deoxycholate and SDS. More studies need to be performed to determine the mechanism of action and provide information about the substrate binding sites of QacE and A1S_0710.

### 2.4. MFS Family Efflux Pumps 

The MFS efflux pumps are a large and diverse family of efflux pumps that are found across all domains of life. They include symporters, antiporters, and uniporters. Together, the MFS family efflux pumps have a diverse range of substrates, but each individual efflux pump within the family is specific to its substrate [8]. Generally, efflux pumps that belong to the MFS category have a similar 3D orientation structure, but proteins in the family can be variable. Some of the proteins have 12 transmembrane segments, while others have 14. The MFS family of efflux pumps uses a proton motive force to export substrates from the cell [94]. Some of these substrates include sugars, drugs, and intermediate metabolites [29]. There are approximately 61 different MFS transporters in the genomes of *A. baumannii* [8].

In *A. baumannii*, the MFS family of efflux pumps includes the TetA, TetB, CraA, CmlA, FloR, AmvA, AbaQ, and EmrAB efflux pumps [8,21,58], and CraA, AmvA, and AbaQ, are known to be present in all strains of *A. baumannii* [8]. Of these pumps, CraA and AmvA are chromosomally encoded, and CraA plays a role in intrinsic resistance [21]. The substrate of CraA is chloramphenicol [51]. It is currently unclear what role CraA plays in clinical isolates of *A. baumannii*, whether CraA is expressed constitutively [8], and if the presence of sodium chloride in the environment causes the overexpression of CraA [95]. AmvA has 14 α-helix transmembrane domains and is under the control of the TetR-type regulator in *A. baumannii* [53]. It has variable substrates, such as erythromycin, and various dyes and disinfectants [54]. 

Some of the efflux pumps that *A. baumannii* can carry and acquire are AbaF, TetA, TetB, CmlA, and FloR [8,21]. TetA efflux pumps, which work in synergy with AdeABC and AdelIJK, allow high levels of resistance to tigecycline and have tetracycline and tigecycline as substrates. The substrates for the TetB pump are minocycline and tetracycline [44,49]. TetA is a trimer consisting of 401 amino acids that make up 12 transmembrane α-helices. It exchanges substrates for a proton. G224 and Q225 within transmembrane helix 7, which is involved in substrate binding, play a role in substrate transport. Transmembrane helices 7 and 9 interact to form a substrate-binding site. Notably, W231, located within transmembrane helix 7, is involved in determining substrate specificity. Leu308, located in transmembrane helix 10, is also thought to play a role in substrate specificity. Substrates bind near a barrier between G224 and A228, which is thought to play a role in the substrate’s movement through TetA. The cytoplasmic loop between transmembrane helices 2 and 3 has a gating function. The transcription of TetA is blocked by the repressor TerR and induced by low levels of tetracycline, which inactivates TetR through reversible binding [96]. In the TetB efflux pump, amino acids at positions 231 and 308 are located in transmembrane domains 7 and 9, respectively, and point toward the center of the efflux pump. These two residues interact with each other and play a role in substrate binding [97]. D23 in the first transmembrane segment, and residue R109 in transmembrane segment 4 in FloR efflux pumps have been found to be necessary for this efflux pump to function. Residues D23 and R109 are involved in the binding pocket and contribute to substrate recognition. It is believed that these two residues work together to orient substrates within the efflux pump [98]. The CmlA, CraA, AmvA, and AbaQ efflux pumps in the MFS family in *A. baumannii* need to be studied further to determine more about their mechanisms of action and their substrate binding sites. The substrates for efflux pumps AbaF, CmlA, and FloR are, respectively, fosfomycin, chloramphenicol, and florfenicol [27,52,55], and their substrates are being studied further. 

The AbaF efflux pump is carried intrinsically in the *A. baumannii* genome, while the Tet transporters are acquired by the transfer of a plasmid or transposons. The CmlA and FloR efflux pumps are found in resistance islands in *A. baumannii* [8,99]. The Tn*1721*-like transposon carries *tetA* and *tetR.* Plasmids that are about 5 to 9 kb carry *tetB*, and the *tetB* gene is associated with IS*CR*2, a plasmid-mediated mobile element [50]. The *tet39* gene, found in *Acinetobacter* spp., and *tetR* are found on about 25 to 50 kb transferable plasmids [100]. In *A. baumannii* AYE, the AbaR1 resistance island (approximately 86 kb) carries the Tn*1721*-like transposon, which harbors *tetA* and *tetR*, and the Aba1 resistance island, *tetG* and *tetR* [52]. The CmlA and FloR efflux pumps are also located on the AbaR1 resistance island in *A. baumannii* AYE. The *floR* and *cmlA* genes are found to be associated with *abaR* in *A. baumannii* chromosomal DNA [29].

**Figure 4:**
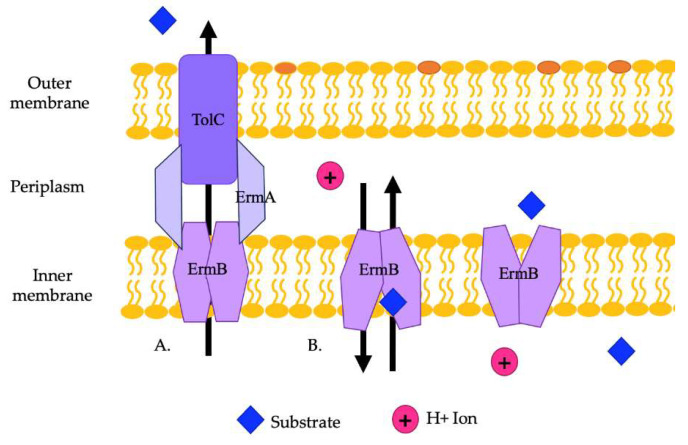
(**A**) A representation of the ErmAB efflux pump and its interaction with TolC in exporting substrates from the cell. (**B**) A representation of the antiporter movements of ErmB. Substrates from the cytoplasm or the inner membrane leaflet are moved by ErmB to the periplasm to be exported by ErmA and TolC.

The EmrAB efflux pump has been found to provide resistance to a variety of antibiotics in various Enterobacteriaceae [58,101]. In *E. coli*, this pump provides resistance to antimicrobial detergents and possibly polymyxin. Some strains of *E. coli* carry the genes *emrA* and *emrB* on plasmids. Studies demonstrated that *A. baumannii* can carry EmrAB efflux pumps, which provide resistance to colistin and polymyxins and play a role in relieving osmotic stress. The ermA and ermB genes are encoded in an operon in *A. baumannii* [59]. Previous studies in *E. coli* showed that the negative regulator of EmrAB is EmrR [57]. ErmAB interacts with TolC to export substrates (Figure 4A). ErmA is a periplasmic adaptor protein that interacts with the OMP, TolC, in a tip-to-tip fashion to create an extended periplasmic canal (Figure 4A). ErmA has β-barrel, lipoyl, and α-helical coiled-coil domains. TolC is a trimer with a periplasmic α-helical tunnel domain and a β-barrel outer membrane channel. ErmB acts as an antiporter and has 14 transmembrane helices. The periplasmic loop of ErmB consists of 53 amino acids located between transmembrane helices 13 and 14. Of note, the periplasmic loop of EmrB is significantly smaller than that of other efflux pumps. It recognizes substrates from the cytoplasm or the inner membrane leaflet (Figure 4B). Substrates are then released on the periplasmic side into ErmA, where they then move through TolC to be exported out of the cell (Figure 4B) [102]. 

### 2.5. ABC Family Efflux Pumps

The ABC family of efflux pumps uses the energy of ATP hydrolysis to pump substrates out of a cell or transport them across a membrane. They have four protein domains, two of which hydrolyze ATP and two of which span the membrane. The domains can all be in the same protein or in multiple proteins. ABC transporters switch between open, occluded, and outward open states to transport substrates out of the cell in a one-way, outward-only fashion [103]. ABC transporters have a highly conserved sequence, and these pumps are found in the domains of Eubacteria, Archaea, and Eukarya [8].

**Figure 5:**
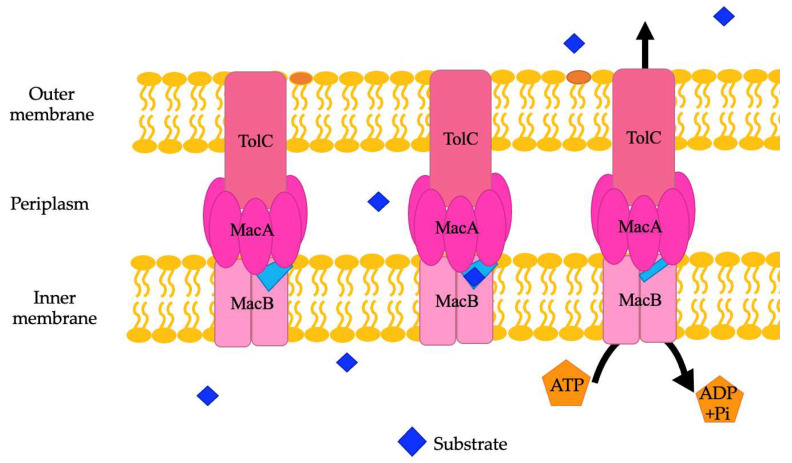
A representation of the MacAB-TolC efflux pump. Within this pump, a light blue periplasmic opening is noted. Substrates may also bind here and be exported through the rest of the efflux pump when coupled conformational changes are caused by ATP hydrolysis.

ABC family efflux pumps are detected in several genera of bacteria, including *Acinetobacter*. There are an average of 94 different ABC efflux pumps found in *A. baumannii* genomes [8]. They need to be further studied in *A. baumannii* and other Gram-negative bacteria to fully establish their exact role in antibiotic resistance. The substrates of MacAB-TolC, an ABC family efflux pump in *A. baumannii* and other *Acinetobacter* species, are erythromycin, gramicidin, and macrolides in other species of bacteria [21,104]. Structurally, the MacAB-TolC efflux pump is different than other ABC family efflux pumps. MacAB-TolC pump is a tripartite complex that includes MacB, an inner membrane protein that functions as a homodimer complex and further consists of two domains, namely, the N-terminal nucleotide-binding domain, which enables power generation via ATP hydrolysis, and the C-terminal cytoplasmic tail. The periplasmic domain of MacB is located between transmembrane helices 1 and 2 and interacts with MacA. MacB has a periplasmic opening that allows for substrate entry from the periplasm, which is different than other efflux pumps in the ABC family (Figure 5). When a substrate enters through this opening, the substrate binding site stays in an outward facing conformation. Conformational changes coupled with ATP hydrolysis cause the binding site to have decreased affinity for the substrate, and then the substrate is pushed through the channel to travel through the remaining portions of the efflux pump and out of the cell [103]. Other components of this system are MacA, a periplasmic adaptor protein that is stimulated when ATPase binds specifically with the lipopolysaccharide core, and the outer membrane channel protein TolC, which functions as an exit duct for substrate transport [105,106]. MacA is a hexamer with a gating ring created by a conserved glutamine ring in the lipoyl domain that acts like a one-way valve during substrate transport [103]. 

TolC is more commonly associated with RND family efflux pumps, is regulated by the BaeSR two-component system and plays a role in resistance to tigecycline [60,61]. Other studies have shown that two other efflux pumps, A1S_1242 and A1S_2622 play a role in antimicrobial resistance, virulence, and motility, and two additional efflux pumps, A1S_0536 and A1S_1535, were found to provide resistance to erythromycin and to chloramphenicol and gentamicin, respectively [15].

### 2.6. PACE Family Efflux Pumps

PACE family efflux pumps are found in many genera of bacteria, most commonly proteobacteria. These pumps are generally found within the core genome of bacteria. Therefore, it is thought that they play other roles than just exporting antimicrobials [65]. The mechanism of action of PACE family efflux pumps is unclear. Both the AceI and A1S_1503 efflux pumps need to be studied further to determine the inhibitor binding site and details of the mechanisms and movements of these efflux pumps. They are predicted to have four α-helices that span across the membrane and lie in two tandem bacterial transmembrane pair (BTP) domains [8]. These proteins are small, and, in the inner membrane, they are thought to act as oligomers. In addition, studies have shown that proton coupling is necessary to move substates across the inner membrane [8].

In the PACE family, the most recently characterized efflux pump in *A. baumannii* is AceI [63,107]. The substrate of the AceI efflux pump is chlorhexidine, a bis-biguanide antimicrobial, which is commonly used in clinical and household settings as an antiseptic in products like soaps, mouthwashes, and lotions for external use. The AceI efflux pump in *A. baumannii* also pumps out short-chain diamines [64]. AceR, a LysR-type regulator, positively regulates the AceI efflux pump when exposed to chlorhexidine [62]. When chlorhexidine is present, it binds to AceR, which can then no longer bind to the DNA. This allows RNA polymerase to bind the DNA and transcribe *aceI*. In addition, another PACE family efflux pump, A1S_1503, which needs to be characterized further, has been found in the *A. baumannii* genome. It provides resistance to acriflavine, a commonly used topical antiseptic [65]. 

### 2.7. Relationship between Efflux Pumps and Virulence

In addition to exporting antibiotics, efflux pumps in *A. baumannii* also contribute to virulence by playing a role in biofilm formation and quorum sensing. Efflux pumps may aid in biofilm formation by exporting quorum quenching molecules and extracellular polymeric substances (EPSs) and by regulating biofilm formation genes [108]. Studies have shown that the AdeABC, AdeFGH, and AdeIJK efflux pumps, which are part of the RND family, play an important role in biofilm formation and maintenance. When these pumps were mutated, there was decreased biofilm formation in comparison to wild type *A. baumannii* [66]. In addition, other studies have found that the AdeABC and AdeIJK pumps are important in regulating pilus system encoding proteins that aid in surface colonization and adhesion, which are important steps in biofilm formation [35]. 

In addition, the AdeFGH efflux pump plays a role in the synthesis and transport of autoinducer molecules that are important in *A. baumannii* biofilm formation [108]. *A. baumannii* uses the AbaI/AbaR two-component system to regulate quorum sensing [109] and *abaM*, which lies between *abaI* and *abaR*, also plays a role in biofilm formation, motility, and virulence in this bacterium [110]. The *abaI* gene codes for autoinducer synthases, which produce AHL. AbaR acts as a receptor for the AHL molecules produced by *abaI.* If quorum sensing is disturbed, there is less biofilm production, and susceptibility to antibiotics is increased [109]. Therefore, if efflux pumps could be blocked by EPIs, the bacterial cells would not only show decreased antibiotic efflux, but there may be decreased biofilm formation. Together, these potential actions of EPIs may make *A. baumannii* infections easier to treat since the cells would be more susceptible to antibiotics for multiple reasons.

## 3. Efflux Pumps Inhibitors

Efflux pump inhibitors (EPIs) provide one potential solution to combat antibiotic resistance. The goal of EPIs is to make resistant bacteria re-susceptible to the desired antibiotic by blocking their efflux pumps [18]. They can do this through a variety of mechanisms, including competitive or non-competitive inhibition, dissipation of the proton gradient needed by efflux pumps to export antibiotics and other substrates [18,21], suppression of the expression of genes that encode efflux pumps, blocking the inner or outer membrane protein used for efflux, disruption of efflux pump assembly, and changing the structure of the medication so that it cannot be recognized. There are numerous compounds, derived from natural and synthetic sources [18], that are known to influence the functioning of efflux pumps, but currently none are clinically approved due to a variety of requirements that must be met to make them clinically successful. More research needs to be carried out to find EPIs that can be clinically successful.

## 4. Known EPIs

**Figure 6:**
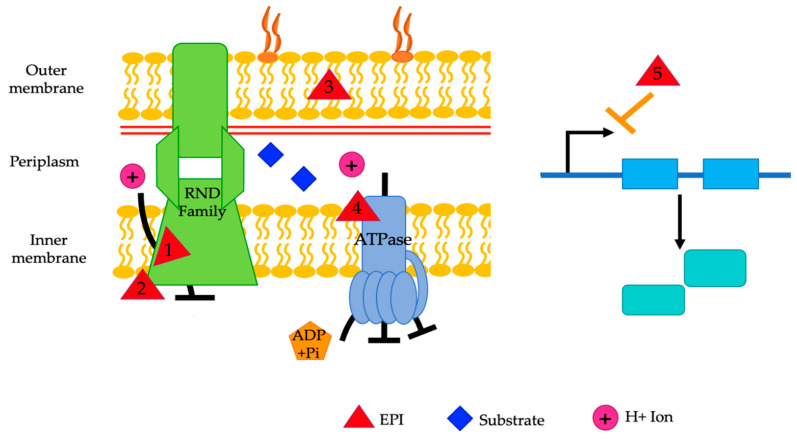
A representative diagram of the mechanisms of action of various EPIs. EPIs work by competitive inhibition (1), non-competitive inhibition (2), altering membrane permeability (3), disruption of the proton motive force (4), and downregulation of gene expression (5). Reserpine, epigallocatechin gallate, PAβN, verapamil, pyridoquinolone, D2, and D13-9001 are theorized to be competitive inhibitors (1). NMP, arylpiperidines, aryl piperazine, and epigallocatechin gallate are believed to act as non-competitive inhibitors (2). PAβN and resveratrol alter membrane permeability (3). CCCP and IITR08027 affect the proton motive force (4). Eugenol, trans-cinnamaldehyde, and resveratrol downregulate gene expression (5). The exact mechanism of action is unknown for the remainder of the mentioned EPIs.

There are a variety of categories that EPIs fall under (Figure 6). Because the mechanism of action is not known for most EPIs, the best way to categorize them is by their source. They can be divided into the categories of plant, synthetic, or microorganism-derived [18]. Nanoparticles, bacteriophages, and non-thermal plasma-treated N-acetylcysteine (NAC) are also being studied for their potential to inhibit efflux pumps [21,111].

### 4.1. Plant-Derived EPIs

#### 4.1.1. Resperine

Reserpine is a plant alkaloid EPI that directly binds efflux pumps, specifically the MFS and RND efflux pumps, in Gram-negative bacteria [18]. It is derived from the roots of the *Rauwolfa serpentina* plant and acts as an antipsychotic medication. In Gram-positive bacteria, it has been shown to directly interact with amino acid residues in efflux pump transporter proteins, specifically Bmr in *B. subtilis*. Reserpine has been shown to increase the susceptibility of *A. baumannii* clinical isolates to levofloxacin [112].

#### 4.1.2. Eugenol and Trans-Cinnamaldehyde

Eugenol and trans-cinnamaldehyde are two naturally derived EPIs that inhibit efflux pumps in *A. baumannii* by downregulating *ade*A and *ade*B, genes in the RND efflux family, and showed restoration of susceptibility to β-lactams [113]. Eugenol is the active component of clove oil (*Eugenia caryophyllus*). Trans-cinnamaldehyde is an aldehyde extracted from the bark of cinnamon (*Cinnamomum zeylandicum*). Both of these compounds are classified as phytochemicals and considered safe by the US FDA [114]. 

#### 4.1.3. Polyamines

Polyamines are another naturally derived EPI (Table 2) that consist of a large category of amino acid-derived metabolites [21,115]. Generally, they have two or more amine moieties connected by aliphatic chains. The most common polyamines are spermidine, spermine, putrescine, and cadaverine. They may play a role in cell growth, biofilm formation, oxidation stress resistance, and nitrogen storage in bacteria. At high concentrations, they can be toxic. Members of the PACE, MFS, and SMR efflux pump families may be involved in polyamine transport [115]. Research suggests that derivatives of polyamines play a role in inhibiting efflux pumps in *A. baumannii* [116], but further investigations are required as a few researchers report that they may be antimicrobial themselves [117]. Polyamines are the substrate of the efflux pump AmvA.

#### 4.1.4. Epigallocatechin Gallate

Epigallocatechin gallate is a plant-derived polyphenol, the flavonoid EPI. In *Campylobacter* spp. and *Staphylococci* spp., it acts on TetK efflux pumps to increase susceptibility to tetracycline, ciprofloxacin, and erythromycin [18]. Epigallocatechin gallate has shown toxicity in studies. In *A. baumannii*, epigallocatechin gallate is reported as effective in restoring antibiotic susceptibility to gentamicin, tetracycline, and cefotaxime [119]. Studies have also shown that epigallocatechin gallate exhibits synergy with cefotaxime and β-lactams, including carbapenems, and acts as an EPI on the AdeABC efflux pump [119]. Alone, it does not show any bactericidal activity.

#### 4.1.5. Resveratrol

Resveratrol (3,5,4′-trihydroxy-trans-stilbene), a phytochemical derived from the skin of red grapes and seeds, is another possible EPI [118,120]. It belongs to the polyphenol stilbenoids group and is a phytoalexin. It has antibacterial properties against various pathogens, decreases membrane integrity, changes bacterial virulence, and prevents biofilm formation by interfering with quorum sensing [118,134]. Resveratrol has been shown to increase the susceptibility of clinical CRAB isolates to chlorohexidine [118]. Alone, resveratrol does not inhibit the growth of *A. baumannii*, but it is believed to act as an inhibitor of AdeB, part of the AdeABC efflux pump, since the expression of AdeB is significantly reduced when resveratrol and chlorohexidine are used together. In addition, resveratrol has been shown to be safe for humans and is used as a preservative in food. Resveratrol also reportedly works as an EPI in *E. coli* [120].

#### 4.1.6. Other Plant-Derived EPIs

*Rosmarinus officinalis*, derived from rosemary and one of its majority compounds, 1,8-cineole (eucalyptol), *Lycopus europaeus*, and the ethanolic extract of *Levisticum officinale*, a perennial herb often used for medicinal purposes and foods, have been shown to act as EPIs in *A. baumannii* and *P. aeruginosa.* They also show synergy with ciprofloxacin [121,135]. There are a few other compounds that exhibit synergy with antibiotics, like many essential oils, and others, like piperine, that seem to work as EPIs in other genera of bacteria. These compounds need further investigation to determine if they work as EPIs in *A. baumannii* [136,137]. 

### 4.2. Synethetic EPIs

#### 4.2.1. PAβN

Phenylalanine-arginine-β-napthylamide (PAβN) is a synthetically derived peptidomimetic (C-capped dipeptide) EPI [18]. It was discovered in 2001 and is recognized as the first EPI [138], but its exact mechanism of action is poorly understood. It may inhibit RND efflux pumps in *A. baumannii* through competitive inhibition, or it may work by altering the membrane permeability of bacterial cells [81,138]. Computational research examining AcrB predicted that PAβN interacts with F_135_, F_178_, F_615_, F_628_, Q_176_, and E_673_ residues [88]. One study has shown that PAβN moves partially out of the binding pocket to straddle the G-loop, reducing the G-loop’s flexibility and blocking movement between the distal and proximal binding pockets [88]. Studies have shown that PAβN, at various concentrations, increases the susceptibility of *A. baumannii* to nalidixic acid, tigecycline, imipenem, chloramphenicol, trimethoprim, and clindamycin [21,81]. Some studies show that it has no effect on colistin, ciprofloxacin, tetracycline, or carbenicillin susceptibility [138,139]. PAβN also functions as an EPI in *P. aeruginosa* at its MexAB, MexCD, and MexEF efflux pumps, which allows for increased susceptibility to levofloxacin, erythromycin, and chloramphenicol in these isolates [138]. PAβN also works on the AcrAB-TolC efflux pumps in *E. coli*, *E. aerogenes*, *K. pneumonia*, and *S. typhimurium* and a variety of homologous efflux pumps in *Campylobacter* spp. [140]. PAβN showed toxicity in mammalian cells [141]. PAβN has two other derivatives, MC-02,595 and MC-04,124, that are under investigation. These compounds show an enhanced stability profile and increased susceptibility of *P. aeruginosa* to levofloxacin [142]. MC-04,124 also shows increased solubility.

#### 4.2.2. NMP

1-(napthylmethyl)-piperazine (NMP) is a synthetically derived EPI and is a derivative of arylpiperazine [18,143]. One study has shown that NMP moves partially out of the binding pocket to straddle the G-loop, reducing the G-loop’s flexibility and blocking movement between the distal and proximal binding pockets [88]. It has been shown to work as an EPI in *E. coli*, acting on AcrAB-TolC and AcrEF efflux pumps, and in *A. baumannii* [18,143]. It is more effective at high concentrations. Studies have shown that NMP inhibits efflux pumps non-competitively by interacting with residue F_610_ in ArcB efflux pumps [123]. NMP has been shown to increase susceptibility to levofloxacin, oxacillin, rifampin, chloramphenicol, and clarithromycin. To a lesser extent, it also increases cells’ susceptibility to fluoroquinolones, azithromycin, clindamycin, nitrofurantoin, and doxycycline [18]. Studies are conflicting about whether NMP increases susceptibility to tigecycline [128,144]. At concentrations four times higher than what is normally used for EPIs, NMP has antibacterial properties [18].

#### 4.2.3. CCCP

Carbonyl cyanide m-chlorophenylhydrazone (CCCP) is a synthetically derived ionophore EPI [18,124]. It inhibits efflux pumps by dissipating the proton motive force so that oxidative phosphorylation is uncoupled. It disrupts both the ΔΨ and ΔpH components of the proton motive force. Disrupting the proton motive force causes bacterial cells to become metabolically inactive. Whether the increased susceptibility to antibiotics when CCCP is used is due to the metabolic inactivity of the cells or whether CCCP truly acts as an EPI is under scientific debate [18]. Since most efflux pumps use the proton motive force to move toxic substrates out of the cell, CCCP inhibits many different families of efflux pumps in *A. baumannii*, including the RND, MATE, SMR, and MFS family efflux pumps [21]. Studies have shown that the use of CCCP can restore susceptibility to tetracycline in *Heliobacter pylori* and *Klebsiella* spp. and increase susceptibility to carbapenems [125,126]. CCCP is more effective at lower concentrations than NMP and PAβN [124,139]. The use of CCCP is limited to the laboratory because it is relatively toxic to mammalian cells [18].

#### 4.2.4. Verapamil

Verapamil is a synthetically derived EPI and calcium channel blocker. Verapamil is used to treat hypertension. It directly inhibits efflux pumps in *Mycobacterium tuberculosis*, making it more susceptible to bedaquiline and ofloxacin [18]. It also has some inhibitory effects in *A. baumannii*, leading to increased susceptibility to tigecycline [128] and competitive inhibition of MATE family efflux pumps [127]. As previously mentioned, verapamil is considered lethal by the US FDA when used with macrolide antibiotics since it can prolong the Q-T interval, leading to an increased risk for hypotension and shock.

#### 4.2.5. Amlodipine

Amlodipine is another synthetically derived EPI and calcium channel blocker [21]. The exact mechanism of action is unknown, but it has been shown to act as an EPI on the AdeABC efflux pump in *A. baumannii* and increases susceptibility to imipenem [129]. It has been found to be more effective than CCCP in increasing susceptibility to imipenem.

#### 4.2.6. IITR08027

IITR08027 is a synthetically derived potential EPI that inhibits the efflux pump AbeM in the MATE family in *A. baumannii* and *E. coli* by dissipating the proton force [130]. It has no antimicrobial activity, and it does not affect the ΔΨ of the proton motive force. It restores the susceptibility of *A. baumannii* to fluoroquinolones and shows low cytotoxicity in studies [130]. Because of its low toxicity, lack of antimicrobial activity, and ability to restore antibiotic susceptibility, it is being further evaluated preclinically.

#### 4.2.7. Quinoline Derivatives

There are a variety of different quinoline derivatives that act on efflux pumps in bacteria [18]. One of these is pyridoquinolone. It acts as a competitive inhibitor for AcrAB-TolC RND family efflux pumps in *E. aerogenes*, which restores susceptibility to norfloxacin [18]. Another set of quinoline derivatives includes 4-substituted thioalkyl, alkylamino, and alkoxyquinolone [18]. These increase the susceptibility of *K. pneumoniae* and *E. aerogenes* to tetracycline, norfloxacin, and chloramphenicol. Another set of quinoline derivatives, which includes 2-phenyl-4(1H)-quinolone and 2-phenyl-4-hydroxyquinoline, inhibits the NorA efflux pump in *S. aureus* [131].

#### 4.2.8. Arylpiperidines and Aryl Piperazine Derivatives

Arylpiperidines and aryl piperazine derivatives include a variety of different EPIs, with NMP being the most widely studied of the group [18]. Arylpiperidine and other derivatives, like 3-arylpiperidines, function as EPIs in *E. coli* and restore susceptibility to linezolid [18]. Phenylpiperidines are selective serotonin reuptake inhibitors that also act as EPIs in *E. coli* at the AcrAB-TolC efflux pump and in *S. aureus* [18]. Arylpiperidines may be toxic to mammalian cells since they can act as serotonin reuptake inhibitors.

#### 4.2.9. Pyridopyrimidine and Pyranopyridine Derivatives

D2 and D13-9001 are derivatives of pyridopyrimidine that act as EPIs by inhibiting the MexAB-OprM efflux pump in *P. aeruginosa* and AcrB in *E. coli* [132]. D13-9001 has been shown to be a competitive inhibitor of MexAb and AcrB [141]. In addition, MBX2319 is another derivative of pyrazolopyridine that acts as an EPI in *E. coli* at the AcrB-TolC efflux pump [123]. It increases the susceptibility of *E. coli* to ciprofloxacin, levofloxacin, piperacillin, and Hoechst dye. Within the AcrB efflux pump, it may cause a ring stacking interaction by interacting with amino acid residues at the hydrophobic trap of this pump.

### 4.3. Microbial Derivative EPIs

In comparison to the other groups of EPIs, there are a small number of EPIs derived from microbes [18]. EA-371α and EA-371δ EPIs are derived from *Streptomyces* spp. They act on the MexAB-OprM efflux pump in *P. aeruginosa* [133]. The EPI 2-(2-aminophenyl) indole, originally isolated from another terrestrial species of *Streptomyces*, IMTB-2501 [145], is now being used as the rational scaffold for chemically synthesizing various indole derivatives to improve efflux pump inhibitory potential for therapeutic use [146]. Similarly, the findings from another microbial realm, *Actinomycetes*, are encouraging, showing high potential for new chemical scaffolds for EPI discovery [147]. Recently, the scaffold of the quinoline class has also been investigated as a microbial EPI [148]. Further studies are required to understand the details of these EPIs.

### 4.4. Other Possible Categories of EPIs

#### 4.4.1. Nanoparticles

Previous studies have shown that nanoparticles have antibacterial activity [21]. Tocopherol polyethylene glycol succinate (TPGS)-capped silver nanoparticles and copper nanoparticles capped with N-lauryltyramine (NLTA) have been shown to act as EPIs [149,150]. Studies have shown mixed results in determining if nanoparticles may be toxic, and further research on this topic is needed [151].

#### 4.4.2. Bacteriophages

Bacteriophages can also potentially inhibit efflux pumps [21]. As previously mentioned, efflux pumps have inner and outer membrane proteins (OMPs). OMPs are formed from β-barrel structures that span across the membrane and have extracellular loops. Phages can bind to these loops and use them as receptors. In response, bacteria may modify or remove the extracellular OMP loops and the expression of other OMP genes may be altered, leading to the inhibition or loss of efflux pumps [21]. This has been shown in studies with *P. aeruginosa* and phage OMKO1 and in studies with E. coli and phages U136B and 132 [21]. In the study with *P. aeruginosa* and phage OMKO1, the phage bound the OMP OprM, which is a component of the MexXY and MexAB efflux pumps [152]. This binding causes the bacteria to modify OprM, which leads to inhibition of the efflux pumps and increases *P. aeruginosa* susceptibility to tetracycline and erythromycin, which are substrates for the MexXY and MexAB efflux pumps [152]. In addition, there appears to be a genetic trade off with the use of phages to treat bacterial infections [152,153]. The bacteria may become more susceptible to antibiotics again as they develop resistance against the phages being used as a treatment. The use of bacteriophages in the treatment of multidrug-resistant *A. baumannii* infections is promising, especially since numerous animal studies show successful results and phage therapy has been used to successfully treat a patient with a multidrug-resistant *A. baumannii* infection in 2017 [154,155]. It is unclear whether the phages used to treat this patient were acting as an efflux pump inhibitor in A. baumannii, as these studies were not performed for the phages used of the strain of A. baumannii causing the infection. Phage therapy presents its own benefits and challenges. More research on phage therapy is needed. 

#### 4.4.3. An Approach to a Combination Product of an Antibiotic and an Antibiotic Potentiator

Recently, the application of an antimicrobial and antimicrobial potentiators has demonstrated success in controlling an infection caused by MDR pathogens during laboratory investigations [156]. Specifically, an efflux pump inhibitor has been shown to potentiate antibiotic activity and restore the antimicrobial agent’s susceptibility. It is predicted to be a promising strategy to combat antimicrobial resistance. Caution is advised for conditions where internal biofilm cells overexpress efflux pumps associated with secondary metabolite extrusion and external bacteria tend to activate pump transporters, mediating antimicrobial resistance. Here, a thorough investigation is crucial to determining the function and substrates of efflux pumps involved in biofilm formation. This research is needed to avoid the accidental induction of biofilms resulting from the misuse of EPIs and antimicrobial agents. Although the studies are in their early phases, EPIs such as PAβN or thioridazine distinctly decreased biofilm formation by up to 80% and appear to be very promising [157]. Recently, some of the U.S. Food and Drug Administration (FDA)-approved drugs showed excellent efflux pump inhibitory activities against clinical isolates of *S. aureus* [158]. These drugs are safer and more promising than the discovery of de novo EPIs, as their pharmacokinetics and biosafety are well known. These include diclofenac, domperidone, and glyceryl trinitrate [159]. Although clinically approved antimicrobial agents show promising results when used in combination with non-antimicrobial approved drugs, in bench-to-bedside investigations, developing a combination product of such drugs might involve challenges during production processing in the pharmaceutical industry.

#### 4.4.4. Non-Thermal Plasma-Treated NAC

Studies have shown that non-thermal plasma-treated NAC solutions have an antimicrobial effect and generate reactive oxygen species (ROS) and reactive nitrogen species (RNS) [111,160]. In studies, this solution was able to inactivate *E. coli* and *A. baumannii* [111,160]. Exposure to oxidation typically leads to a scavenging effect by bacterial cells and an alteration in cellular metabolic activities, membrane-associated porins, and efflux pumps [161]. Efflux pumps play an essential role in different stress environments for bacteria and, thus, can be a promising target for developing new inhibitors [107]. Enhancing antimicrobial activity has been shown through the synergistic effects of non-thermal (cold) plasma and secondary metabolites [161]. Control studies are required to determine the exact mechanism of action of ROS and RNS in non-thermal plasma-treated solutions and their interactions with antibiotic resistance-mediated efflux pumps. 

### 4.5. Criteria for Successful Efflux Pump Inhibitors

There are strict requirements a compound must meet to be a successful EPI that can be used clinically [14,107]. The compounds cannot have intrinsic antibacterial activity, or else the bacteria can eventually develop resistance, and the molecule will no longer be useful as an EPI [162]. Although the compound should target bacterial cell structures, many times this requirement is challenging to meet since both eukaryotic and prokaryotic cells have efflux pumps wherein they partially share structural similarity. Therefore, inhibition can often impact both efflux pumps [14,163]. In addition, there are certain pharmacological requirements that should be met. Some of these requirements include being non-toxic, having high safety and therapeutic indices, having serum stability, and having a good ADMET (Absorption, Distribution, Metabolism, Excretion, and Toxicity) profile [14,18]. Lastly, the production of the compound must be economically feasible for it to be successfully commercialized [14,18]. Based on the challenges in pharmacological and toxicological testing, production, and marketing, further discussed below, we speculate that having a dual EPI/antibiotic compound would be advantageous. Specifically, this would decrease the regulatory burden associated with the monotherapy route to market, which is easier in dual-compound pharmacokinetic profiling.

### 4.6. Challenges and Potential to Developing Successful Efflux Pump Inhibitors

There are many challenges in developing new EPIs. Clinically, no candidate compound has been approved for use as an efflux pump inhibitor [18,21]. A few medications that have been approved for other indications, such as amlodipine, have been tested and shown to work as EPIs, but these have not undergone further clinical testing and, therefore, have not been approved for use as an EPI. There are few candidate EPIs that have passed through preliminary clinical testing, but further investigations are awaiting [132,138]. So far, most studies of EPIs have only been performed in a laboratory setting to examine their effect on the activity of efflux pumps in bacteria. Synthetic EPIs present a variety of challenges. They are simple to synthesize, but they often have high toxicity, poor cell permeability, and poor solubility [164]. 

Pharmacokinetic issues present another challenge to the use of EPIs. EPIs must be used in combination with antibiotics because they only allow antibiotics to accumulate in the cell and do not directly kill the bacteria. Sometimes this can lead to unwanted interactions and outcomes. For example, verapamil, a nondihyropyridone calcium channel blocker that is also considered an EPI, was tested with macrolide antibiotics like erythromycin and clarithromycin [165]. It was later considered lethal by the US FDA because the combination use of macrolides and verapamil prolongs the Q-T interval and results in a greatly increased risk of shock and hypotension [165,166]. In addition, clarithromycin acts at the same cytochrome that controls the metabolism of verapamil, which can lead to the accumulation of verapamil at toxic levels. Further testing is needed to learn more about the pharmacokinetics of combined EPI and antibiotic use since most testing of EPIs has only been performed in a laboratory setting where potential negative interactions are generally not considered. Also, we suggest an inherent need to match the pharmacokinetic profiles of both the EPI and antibiotic since they need to work at the same location in the body.

Another challenge to the development of EPIs is the collection of clinical and preclinical data, which needs model organisms to test the EPIs [18]. So far, there is limited research on model organisms to test EPIs, and there is limited data from patients to show that EPIs can be successfully used. So far, *C. elegans*, *G. melonella*, *D. melanogaster*, and zebrafish have been successfully used as model organisms to study various efflux pumps, while murine models showed mixed results [140,167,168,169,170].

Additionally, depending on the mechanism of action, bacteria may become resistant to the EPIs [18]. For example, with EPIs that bind directly to the efflux pump, bacteria may mutate these sites so that the EPI is no longer able to bind, thus making that EPI ineffective [18]. Another challenge in the development of EPIs is the fact that bacteria often carry multiple genes that allow for antibiotic resistance, not just genes for efflux pumps. If a bacterium carries more resistance genes than just the gene for the efflux pump, it is unclear if the EPI would still be effective in allowing the bacteria to become susceptible to the desired antibiotic again. For example, *A. baumannii* can carry genes for carbapenemases and the overexpression of efflux pumps, both of which contribute to its resistance to carbapenem [21]. Additionally, the EPIs do not work for all substrates of a specific efflux pump. For example, PAβN enhances the effect of some antibiotic substrates of the MexAB efflux pump in *P. aeruginosa* but not other antibiotics that are substrates of the same pump [21].

Despite these challenges, there has been increased interest in EPIs by pharmaceutical companies recently [21]. Some are working to develop anti-resistance drug candidates that will enable the use of a variety of antibiotics against multidrug-resistant organisms such as ESKAPE pathogens (*E. faecium*, *S. aureus*, *K. pneumoniae*, *A. baumannii*, *P. aeruginosa*, and *Enterobacter* species) [171]. During investigations, it has been shown that their EPIs are effective in increasing bacteria’s susceptibility to sulfonamides, fluoroquinolones, tetracyclines, antimycobacterial agents, monobactams, cephalosporins, and macrolides, and these EPIs showed synergy with 28 antibiotics that were considered less or ineffective. Further clinical investigations are underway. Both broad-spectrum potential EPIs and pathogen-specific potential EPIs are being investigated.

## 5. Conclusions

So far, efflux pumps from the RND, MATE, SMR, MFS, ABC, and PACE families have been shown to play a role in exporting antibiotics in *A. baumannii*. More research needs to be performed to further characterize the various efflux pumps within each family so that their exact physiological roles and roles in multidrug resistance can be determined. This research can provide vital information that can aid in the development of novel EPIs, increase understanding of and correlate mechanisms of action, and slow the rise of antibiotic resistance. In addition, many plant-, synthetically, and microbially derived EPIs have shown the potential to work as EPIs in *A. baumannii*. More research needs to be carried out on these EPIs to see how they perform clinically. Also, taking an approach to broad-spectrum EPI would be scientifically exciting. Despite the challenges, EPIs still offer an additional, promising solution to curb the spread of antibiotic resistance. The development of EPIs that can be used clinically would extend the life of current antibiotics and could potentially allow clinically approved antibiotics to be used at lower doses so that they are less toxic. EPIs from natural sources and chemically synthesized EPIs both hold promise in the restoration of antimicrobial susceptibilities and, therefore, in the management of multidrug-resistant organisms that cause fulminating infections. Further clinical studies are required for EPIs to be used clinically.

## Figures and Tables

**Table 2:** EPIs.

Category	EPI	Derived from	Acts on	Increases Susceptibility to
Plant derived	Reserpine	Roots of Rauwolfa serpentina [18]	MFS and RND family efflux pumps [18]	Levofloxacin [112]
	Eugenol	Clove oil [114]	RND family efflux pumps [113]	β-lactams [113]
	Trans-cinnamaldehyde	Bark of cinnamon [114]	RND family efflux pumps [113]	β-lactams [113]
	Polyamines	Amino acid-derived metabolites [115]	PACE, MFS, and SMR family efflux pumps [115]	Unknown
	Epigallocatechin gallate	Plant-derived polyphenol, flavonoid [118]	AdeABC efflux pump [119]	Gentamicin, tetracycline, cefotaxime, and β-lactams (carbapenems) [119]
	Resveratrol	Skin of red grapes and seeds [118,120]	AdeABC efflux pump [118]	Chlorohexidine [118]
	Rosmarinus officinalis	Rosemary [121]	Unknown	Ciprofloxacin [121]
	Lycopus europaeus		Unknown	Ciprofloxacin [121]
	Levisticum officinale L		Unknown	Ciprofloxacin [121]
Synthetically derived	PAβN	Peptidomimetic (C-capped dipeptide) [18,122]	AcrB efflux pump [88]	Nalidixic acid, tigecycline, imipenem, chloramphenicol, trimethoprim, and clindamycin [21,81]
	NMP	Arylpiperazine [18]	RND family efflux pumps, AcrAB and AcrEF efflux pumps [18,123]	Levofloxacin, oxacillin, rifampin, chloramphenicol, and clarithromycin (lesser extent: fluoroquinolones, azithromycin, clindamycin, nitrofurantoin, and doxycycline) [18]
	CCCP	Ionophore [18,124]	RND, MATE, SMR, and MFS family efflux pumps [21]	Carbapenems [125,126]
	Verapamil		MATE family efflux pumps [127]	Tigecycline [128]
	Amlodipine		AdeABC efflux pump [129]	Imipenem [129]
	IITR08027 [130]	Unknown	AbeM efflux pump [130]	Fluoroquinolones [130]
	Pyridoquinolone	Quinoline derivative [18]	AcrAB-TolC and RND family efflux pumps [18]	Norfloxacin [18]
	4-substituted thioalkyl alkylamino and alkoxy quinolone	Quinoline derivatives [18]	Unknown	Tetracycline, norfloxacin, and chloramphenicol [18]
	2-phenyl-4(1H)- quinolone and 2-phenyl-4-hydroxyquinoline	Quinoline derivatives [131]	NorA efflux pump [131]	Unknown
	Arylpiperidines and aryl piperazine		Unknown	Linezolid [18]
	Phenylpiperidines	Arylpiperidines and aryl piperazine [18]	AcrB-TolC efflux pump [18]	Unknown
	D2 and D13-9001	Pyridopyrimidine [132]	MexAB-OprM and AcrB efflux pumps [132]	Unknown
	MBX2319	Pyrazolopyridine [123]	AcrB-TolC efflux pump [123]	Ciprofloxacin, levofloxacin, piperacillin, and Hoechst dye [123]
Microbially derived	EA-371α [133]	*Streptomyces* spp. [133]	MexAB-OprM efflux pump [133]	Unknown
	EA-371δ [133]	*Streptomyces* spp. [133]	MexAB-OprM efflux pump [133]	Unknown

## Data Availability

No additional data; all data were submitted in this review.
